# The Establishment of an Antiviral State by Pyrimidine Synthesis Inhibitor is Cell Type-Specific

**Published:** 2015-11-06

**Authors:** Donghoon Chung

**Affiliations:** Department of Microbiology and Immunology, Center for Predictive Medicine, School of Medicine, University of Louisville, USA

## Introduction

The replication of virus is dependent on the host metabolism; nucleotide precursors for the synthesis of viral genome or viral mRNAs are supplied from the host nucleotides pool. As viral replication requires a massive synthesis of viral mRNA or viral genome, the inhibition of cellular nucleotide synthesis is considered a strategy for broad-spectrum antivirals. Indeed, the inhibition of GTP synthesis showed antiviral effects against many viruses. Mycophenolic acid (MPA) has broad-spectrum antiviral activity and the mechanism is to decrease cellular GTP concentration by inhibiting inosine-5’-monophosphate dehydrogenase (IMPDH), which catalyzes the rate-limiting reaction of de novo GTP biosynthesis [1]. Many IMPDH inhibitors, however, show noticeable cytotoxicity at the effective concentrations, making them less attractive as an antiviral therapeutic [2–5].

**Figure 1:**
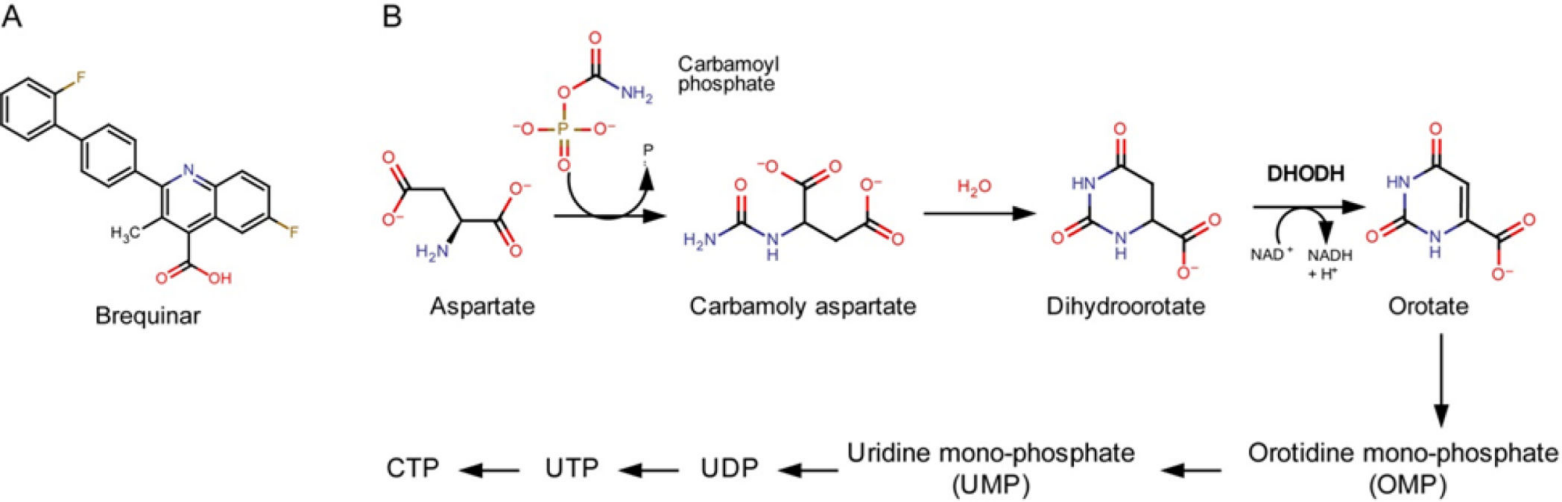
Brequinar and DHODH, Structure of brequinar (A), and the pyrimidine de novo biosynthesis pathway (B).DHODH is the rate limiting step and inhibited by brequinar.

On the other hand, pyrimidine synthesis inhibitors are getting more attention as a novel antiviral strategy. Several pyrimidine synthesis inhibitors have been discovered as active hit compounds from high-throughput screenings for antivirals [6–8]. Unlike MPA, these pyrimidine synthesis inhibitors did not show toxicity to the cells at the effective concentrations. Recently, a novel antiviral mechanism was discovered that could interpret the robust antiviral activity of pyrimidine synthesis inhibitors. Marianne et al. has shown that brequinar or DD264, a dihydroorotate dehydrogenase (DHODH) inhibitor, has broad-spectrum antiviral activity and the treatment of the cells with the compounds induced the expression of IFN-stimulated genes (ISGs) that are associated with the antiviral effects [9]. The compound decreased cellular pyrimidine concentration; however, the decrease of pyrimidine concentration was not the main antiviral mechanism. More importantly, the antiviral effect was dependent on the synthesis of new proteins under the control of interferon regulatory transcription factor 1 (IRF1). This finding clearly illustrates how pyrimidine synthesis inhibitors could exert potent broad-spectrum antiviral activity without cytotoxicity unlike MPA. This finding could lead to the development of broad-spectrum antivirals from pyrimidine synthesis inhibitors. In support of the mechanism, leflunomide, an immunosuppressant drug that inhibits DHODH, the fourth enzyme of the pyrimidine biosynthesis (Figure 1), has been reported to have an antiviral effect against several viruses in a clinical study [10].

Despite this prominent antiviral effect in vitro, none of the pyrimidine synthesis inhibitors have shown antiviral effect in vivo models using mice [6–8]. For this reason, pyrimidine synthesis inhibition has not been accepted as a viable antiviral strategy. It has been speculated that the concentration of exogenous pyrimidines in the serum is too high to inhibit viral replication. This argument, however, can’t explain the lack of antiviral effect in mice completely. Wang et al. showed about a 50% decrease in uridine levels in mice treated with their compound, NITD-982 [8]. With the decrease in the pyrimidine concentration, the induction of ISGs was expected after the treatment of the mice, which could lead to an antiviral activity. As mentioned earlier, no antiviral effect was observed in various in vivo models, which is contradicting to the clinical finding with leflunomide.

In this study, we sought to understand better why pyrimidine synthesis inhibitors are not successful in inhibiting virus replication in mouse models. During the study of a novel pyrimidine inhibitor as a broad-spectrum antiviral, we observed results that are similar to Marianne et al. in that cells treated with pyrimidine synthesis inhibitors reduced virus replication significantly. More interestingly, we found that such antiviral effect was cell line-specific: i.e., human cell lines established an antiviral state by the treatment of pyrimidine synthesis inhibitors, but mouse cell lines did not. This observation could explain the lack of antiviral effect of pyrimidine synthesis inhibitors in mouse models. This finding may imply the fundamental difference in the mechanism of innate immune system in response to the inhibition of pyrimidine biosynthesis between human and mouse.

## Results

### Antiviral activity of brequinar and monensin

To test whether pyrimidine synthesis inhibitors can inhibit virus replication in mouse cells in vitro, we measured the antiviral activity of brequinar and monensin in several cell lines through determining an EC_50_ for each. Monensin inhibits the acidification of endosome, which is required for the viruses to infect and release the genetic materials into the cytoplasm [11]. Brequinar is a well-known pyrimidine synthesis inhibitor (Figure 1) and has been tested for cancer treatment in Phase I and II trials [12]. The cell lines we tested include HEK 293T (human embryonic kidney), SY-SH5S (human bone marrow derived neuroblast), Vero76 (African green monkey kidney fibroblast), BHK C21 (hamster kidney fibroblast), Neuro2A (mouse neuroblast), and NIH3T3 (mouse fibroblast). For measurement of antiviral activity, two recombinant viruses, V3526-luc (alphavirus) and pseudotyped ΔG-luciferase (pVSV-luc, rhabdovirus), were used. V3526-luc was constructed by inserting a firefly luciferase gene under viral nonstructural protein 3 of Venezuelan equine encephalitis virus strain V3526 [13]. pVSV-luc is a non-replicating pseudovirus with homologous viral Gp proteins from Vesicular stomatitis virus [14].

The antiviral activities of brequinar and monensin in various cell lines are summarized in Table 1 as a format of EC50, half-maximal effective concentration. The antiviral activities of monensin were very close to each other in all cell lines tested in this experiment. The EC50s were within a range between 0.08 and 0.25 for V3526-luc, and between 0.17 and 0.56 µM for pVSV-luc. The maximum fold-difference of EC50s was less than 3.3 (EC50 in SY-SH5S compared to that in Vero76). This result shows that endocytosis is a critical pathway for the viruses in the cell lines and monensin worked equally in the cell lines.

In contrast to monensin, the antiviral activities of brequinar were cell line-specific. The EC50s of brequinar in 293T cells (human) were 0.031~0.039 µM for the viruses, implying a strong antiviral activity against both viruses in these cell lines. The antiviral activities in murine cell lines we tested (BHK, Neuro 2A and NIH 3T3), however, were much less compared to in 293T cell lines. The EC_50_s were ~1 µM for both viruses, which is around 30-fold higher than those in 293T cells. Interestingly, brequinar did not show a strong antiviral activity in SY-SH5S cell line with an EC_50_ of 1.13 µM, even though it is a human cell line. These data clearly indicate that antiviral activity of brequinar is much less efficient in mouse cells and is cell line-dependent. These responses were the same with other stains of VEEV, such as V3526 or TC-83 (data not shown).

### Induction of antiviral response gene by brequinar

**Figure 2:**
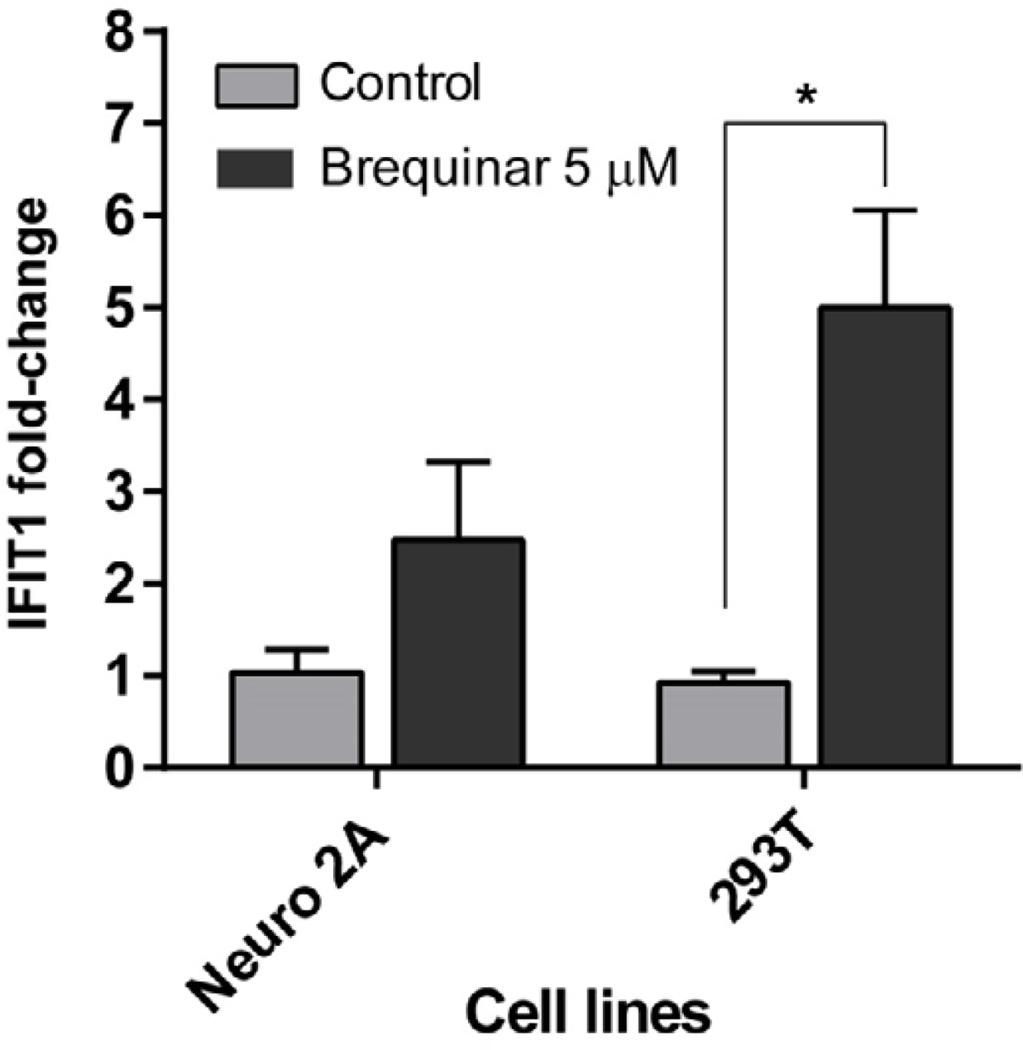
Induction of IFIT1 by brequinar in 293T and Neuro 2A cells. Total RNAs from cells in a 6-well plate were isolated with RNAzol® RT (Molecular Research Center, Inc) reagent as per the manufacturer’s protocol and were dissolved in 50 µL of THE RNA Storage Solution (Life Technologies). One microgram of RNA samples were subjected to a cDNA synthesis with Maxima™ HMinus Reverse Transcriptase (Life Technologies), random hexamers, and oligo-dT by following the manufacturer’s protocol. The relative amount of IFIT1 was calculated using a real-time PCR assay with 2(−Delta DeltaC(T)) method in a total of twenty microliters per well with 2 µL of 2-fold diluted cDNA mixture in a multiplex mode in conjunction with TaqMan chemistry. Human GAPDH (Life Technologies Cat. 4326317E) and mouse GAPDH (Cat. 4352339E) primer probe mix were used as the endogenous controls and human IFIT1 (Life Technologies assay ID: Hs01911452_s1) and mouse IFIT1 (Life Technologies assay ID: Mm00515153_m1) were used to quantitate the RNA copy numbers. Three biological replicates along with two technical replicates were used for the quantitation.

To test whether the cell type-dependent antiviral activity of brequinar is associated with the induction of ISGs, we measured the induction of Interferon-Induced Protein With Tetratricopeptide Repeats 1 (IFIT1) gene as a marker for ISGs in two cell lines; 293T (responsive to brequinar) and Neuro 2A (less responsive to brequinar) after a treatment with brequinar for 18 hours (Figure 2). Treatment of brequinar increased IFIT1 gene expression significantly (4-fold increase, P=0.02 by Student t-test). However, this increase in IFIT1 in Neuro2A cells was not high as in 293T. The expression of IFIT1 was increased by 1.45-fold and was not statistically significant (P=0.09 by Student t-test). This result shows that brequinar is less efficient in inducing ISGs in mouse cells, which is consistent with our antiviral assay result (Table 1).

## Discussion

The antiviral activity associated with pyrimidine synthesis inhibition is expected to depend on two mechanisms: 1) direct effect from decreased pyrimidine concentrations for viral RNA and DNA synthesis, and 2) indirect effect through the induction of ISGs. It is difficult to measure the sole impact of each mechanism on the viral replication, as the latter mechanism is dependent on the first mechanism. The silencing of IRF1, which induces the ISGs, abrogated antiviral effect of brequinar; hence, the second mechanism seems more important for the antiviral activity [9].

The lower antiviral activity of brequinar that was observed in mouse cells could be due to the binding specificity to mouse DHODH. All mammalian DHODHs are very similar and clustered in Family 2 group. The sequence identity between mouse and human DHODH is 88% [15]. However, the binding site of brequinar is known to be species-specific; brequinar has less binding affinity to rat DHODH compared to human’s [16]. There is no known study with mouse DHODH and its inhibitors. As the sequence identity between rat (GenBank Ref ID, NP_001008553.1) and mouse is 95% (GenBank Ref ID, NP_064430.1), it is expected that brequinar is less efficient to mouse DHODH. But a study with a mouse line showed brequinar was able to decrease UTP and CTP up to 4% of their initial levels, which could suggest the decrease in pyrimidine concentration may not be the determining factor for the decrease in antiviral activity observed in mouse cells [17]. Similarly, hamster cell line (BHK) was not responsive to the treatment of brequinar either (Table 1). Considering mouse and hamster share the sequences with a sequence identify of 96% (GenBank Ref ID, ERE78904.1), the antiviral activity in hamster cells might be due to the genetic similarity of the DHODHs. 293T (human cell line) and Vero 76 (African green monkey cell line) showed similar antiviral response upon the treatment of brequinar.

An alternative hypothesis could be an absence of a pathway to activate ISGs in response to decreased cellular pyrimidine concentration. While both SH-SY5S and 293T cell lines are human cell lines, the antiviral activity of brequinar in the two cell lines were clearly different (~30-fold difference). Experiments with other pyrimidine synthesis inhibitors showed a lack of antiviral activity in the mouse cell lines (data not shown). The absence of an antiviral effect in mice regardless of their structure suggests a difference downstream pathway is involved. Currently, the pathway is unknown and we are currently focused on identifying of the nature of the pathway in both human and mouse.

Aside from the mechanism, this study sheds light on selecting a proper animal model to evaluate antiviral activity of pyrimidine synthesis inhibitors. Many in vivo antiviral efficacy models use mice as the first line model for the convenience and virus susceptibility [18–20]. In fact, the animal models in which all pyrimidine synthesis inhibitors have been evaluated for their antiviral activities were mice or cotton rat models, and no antiviral activity has been observed [6–8,21]. Based on our study showing greatly reduced antiviral activity in mouse cell lines, mouse models should be avoided for testing this class of compounds in vivo. Rather, the non-human primate model could be considered for the compounds. We are currently working on establishing an animal model to evaluate the antiviral activity of this class of inhibitors, as well as novel developing pyrimidine synthesis inhibitors that are active in human cells in addition to other small animal cells. A successful outcome will allow us to develop novel broad-spectrum antiviral therapeutics from a pyrimidine synthesis inhibitor inducing ISGs and establishing antiviral states.

## Figures and Tables

**Table 1:** Antiviral activity of brequinar and monensin in various cell lines.

	EC_50_ (µM) of			
	Monensin		Brequinar	
	V3526-luc	pVSV-luc	V3526-luc	pVSV-luc
**293T**	0.08	0.19	0.039	0.031
**SY-SH5S**	0.02	0.17	1.13	3.712
**Vero76**	NT	0.56	0.094	0.303
**BHK**	0.092	0.503	0.903	>25
**Neuro2A**	0.25	0.22	0.967	1.03
**NIH 3T3**	0.02	0.244	0.765	0.932

To test antiviral effects, EC_50_ were evaluated in a dose-response format starting from 25 µM by a five-fold dilution, triplicates for each, in a 96-well format. Cells were suspended in a cell culture media and seeded in white well plates in a volume of 45 microliters and incubated in an actively humidified incubator with 5.0% CO_2_ at 37°C and 95% humidity for 18 hours. Test compounds diluted in thirty microliters of cell culture medium was added to each well. After a two-hours incubation at 37°C with a 5.0% CO_2_, virus was added to the wells in a volume of fifteen microliters then incubated 18 hours further. The luciferase activity was measured with Bright-Glo™ reagent (Promega). The assay conditions were optimized for each cell line. 293T, Vero76, Neuro 2A, and BHK cells were maintained in Minimum Essential Medium with Earl's modification (MEM-E) containing 10% fetal bovine serum (FBS) and 1X GlutaMAX (Gibco 35050-061). NIH 3T3 and SY-SH5S cells were maintained in Dulbecco's modified Eagle's medium with 10% FBS or MEM-E/F12 medium with 10% FBS, respectively. For assays with 293T, Neuro 2A, and SH-SY5Y cells, 24,000 cells and 2,400 TCID_50_ units of virus per well was used. For Vero 76 and BHK cells, 12,000 cells and 1200 TCID_50_ units of virus per well was used. For NIH3T3, 24,000 cells and 20,000 TCID50 units of virus per well was used. IC_50_s were calculated using XLfit (IDBS) formula 205, a 4-parameter Levenburg-Marquardt algorithm with maximum and minimum limits set at 100 and 0, respectively.
